# β-Carotene Supplementation Improves Pancreas Function during Moderate Ethanol Consumption: Initial Characterization from a Morphological Overview

**DOI:** 10.3390/ijms25021219

**Published:** 2024-01-19

**Authors:** Cristian Sandoval, Angeles Vera, Katherine Birditt, Karina Godoy, Florencia Carmine, José Caamaño, Jorge Farías

**Affiliations:** 1 Escuela de Tecnología Médica, Facultad de Salud, Universidad Santo Tomás, Los Carreras 753, Osorno 5310431, Chile; cristian.sandoval@ufrontera.cl; 2 Departamento de Ingeniería Química, Facultad de Ingeniería y Ciencias, Universidad de La Frontera, Temuco 4811230, Chile; 3 Departamento de Medicina Interna, Facultad de Medicina, Universidad de La Frontera, Temuco 4811230, Chile; 4 Carrera de Tecnología Médica, Facultad de Medicina, Universidad de La Frontera, Temuco 4811230, Chile; a.vera08@ufromail.cl; 5 Physiology Development and Neuroscience Department, University of Cambridge, Cambridge CB2 1TN, UK; krb56@cam.ac.uk; 6 Núcleo Científico y Tecnológico en Biorecursos (BIOREN), Universidad de La Frontera, Temuco 4811230, Chile; karina.godoy@ufrontera.cl; 7 Carrera de Medicina, Facultad de Medicina, Universidad de La Frontera, Temuco 4811230, Chile; f.carmine02@ufromail.cl; 8 Laboratorio de Inmunohematología y Medicina Transfusional, Departamento de Medicina Interna, Facultad de Medicina, Universidad de La Frontera, Temuco 4811230, Chile

**Keywords:** alcohol intake, alcohol pancreatitis, antioxidant treatment, chronic alcohol consumption

## Abstract

Alcohol is believed to harm acinar cells, pancreatic ductal epithelium, and pancreatic stellate cells. After giving ethanol and/or β-carotene to *C57BL/6* mice, our goal was to evaluate their biochemistry, histology, and morpho-quantitative features. There were six groups of *C57BL/6* mice: 1. Group C (control), 2. Group LA (low-dose alcohol), 3. Group MA (moderate-dose alcohol), 4. Group B (β-carotene), 5. Group LA + B (low-dose alcohol combined with β-carotene), and 6. Group MA + B (moderate-dose alcohol combined with β-carotene). After the animals were euthanized on day 28, each specimen’s pancreatic tissue was taken. Lipase, uric acid, and amylase were assessed using biochemical assessment. Furthermore, the examination of the pancreatic structure was conducted using Ammann’s fibrosis scoring system. Finally, the morpho-quantitative characteristics of the pancreatic islets and acinar cells were determined. In the serum of the MA + B group, there were higher amounts of total amylase (825.953 ± 193.412 U/L) and lower amounts of lipase (47.139 ± 6.099 U/L) (*p* < 0.05). Furthermore, Ammann’s fibrosis punctuation in the pancreas revealed significant variations between the groups (*p* < 0.001). Finally, the stereological analysis of pancreatic islets showed that the groups were different (*p* < 0.001). These findings suggest that antioxidant treatments might help decrease the negative effects of ethanol exposure in animal models.

## 1. Introduction

The use of alcohol is a significant issue for public health. Based on findings from the United European Gastroenterology Survey of Digestive Health, 155 billion euros per year are spent as a consequence of alcohol intake [1]. The use of alcohol is a well-recognized risk factor for both acute and chronic pancreatitis, accounting for around 50 to 80% of all reported cases [2]. Recent studies have reported that alcohol remains the primary cause of this condition in the United States [3].

In contrast, it has been shown that fewer than 5% of heavy alcohol drinkers experience pancreatitis [4]. The main reason is that showing signs of alcohol toxicity illness requires the presence of other risk factors, which may be caused by the environment or be inherited [5,6].

Acute pancreatitis is a pathological illness defined by the inflammatory response of the pancreas, which can result in local tissue destruction, a systemic inflammatory response, and, ultimately, organ failure [7]. The incidence of this gastrointestinal illness is widespread, and it necessitates prompt hospitalization [8,9].

According to the American College of Gastroenterology guidelines, alcohol can be attributed as the etiological factor for acute pancreatitis when a patient has a documented history of consuming alcohol excessively for a duration beyond five years (>50 g/per day) [10]. But, in some instances, the etiology of acute pancreatitis can be attributed to alcohol drinking when the consumption is infrequent or moderate [11].

The intricate and multifaceted effects of alcohol on the pancreas have left the etiology of alcohol-induced pancreatitis unclear. Alcohol is believed to damage acinar cells, pancreatic ductal epithelium, and pancreatic stellate cells, thereby promoting pancreatic fibrosis [12,13]. Alcohol-induced pancreatitis only occurs when compensatory mechanisms are exhausted or when there is an increased susceptibility to other (genetic and environmental) pancreatic stressors. However, animal models indicate that the pancreas can mitigate alcohol’s detrimental effects through an adaptive stress response [14,15].

Pancreatic acinar cells use both oxidative and nonoxidative pathways to metabolize alcohol [16,17,18]. In fact, previous studies have demonstrated that alcohol probably alters the class II alcohol dehydrogenase (ADH2) [19] and ADH3 isoforms [19,20] to inhibit acinar ethanol oxidation. Some of the bad things that alcohol does to acinar cells are because of how it is broken down and how it makes toxic compounds like fatty acid ethyl esters (FAEEs), acetaldehyde, and reactive oxygen species (ROS) [16,21,22]. Oxidative stress, which is caused by ROS and FAEE, leads to destabilization of zymogen granules and lysosomes and other dysregulations of the cell organelles [4,16,23].

The process of oxidative alcohol metabolism leads to impairment of mitochondrial activity, which serves as a stimulus for the initiation of apoptosis and necrosis [24]. Mitochondrial dysfunction arises due to the permeabilization of membranes caused by oxidative alcohol metabolism [25].

The dysfunction of autophagy is also a prominent symptom of alcohol-induced pancreatitis. The process of autophagy commences by sequestering the material that is intended for destruction into autophagosomes. These autophagosomes subsequently merge with lysosomes, resulting in the formation of autolysosomes. Within these autolysosomes, the cargo undergoes disintegration facilitated by lysosomal hydrolases [26]. One of the early indications of pancreatitis is the presence of acinar cells that have larger autolysosomes, which carry poorly digested cargo [27]. Experimental models of alcohol-induced pancreatitis have shown a considerable drop in levels of lysosome-associated membrane proteins, which play a critical role in preserving the functionality of lysosomes [28,29].

Impairment of the apical secretion of zymogens is an additional significant mechanism that impacts acinar cells and plays a role in the development of alcohol-induced pancreatitis. A block of apical secretion triggers the activation of proteases inside the acinar cells, as well as the release of active zymogens into the interstitial space by basolateral exocytosis via the basolateral plasma membrane of the acinar cell [30,31,32].

The pathophysiology of alcohol-induced pancreatitis has been described in previous research. Despite this, there is still a lack of conclusive comprehension of this significant issue. Therefore, the aim of this study was to evaluate the biochemical, histological, and morpho-quantitative effects of oral supplementation with β-carotene on the pancreas of *C57BL/6* mice exposed to ethanol consumption.

## 2. Results

### 2.1. Biochemical Evaluation

Biochemical analyses for lipase, uric acid, and amylase are shown in Table 1. Briefly, the LA group showed the lowest lipase activity among the experimental groups, whereas the B group showed the highest lipase activity (*p* = 0.002). In relation to uric acid levels, the MA group presented the lowest levels and the LA group the highest levels (*p* < 0.001). Moreover, no significant differences between groups were found in amylase activity (*p* = 0.155).

### 2.2. Histological Evaluation

**Figure 1:**
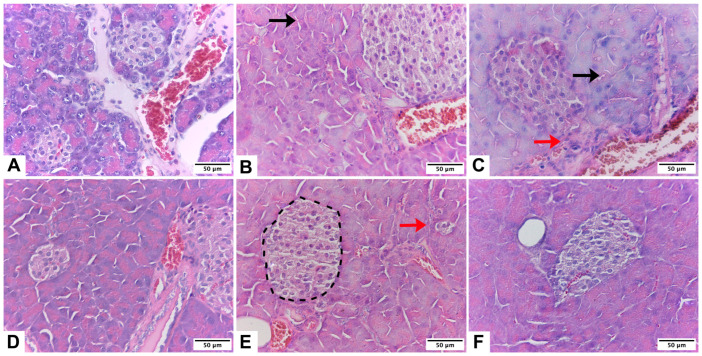
Pancreas of male *C57BL/6* mice. Pancreatic architecture was observed in groups C (**A**), low-dose alcohol (**B**), moderate-dose alcohol (**C**), β-carotene (**D**), low-dose alcohol + β-carotene (**E**), and moderate-dose alcohol + β-carotene (**F**). Interlobular fibrosis (black arrow), perilobular fibrosis (red arrow), and pancreatic islet (segmented line).

As depicted in Figure 1A, pancreas cells in the control group possessed large nuclei surrounded by a well-defined cytoplasm and cytoplasmic membrane; they also exhibited normal morphology of the islets. The islet appeared less stained than the surrounding acinar cells and was composed of polygonal cells arranged in bundles and separated by a network of blood vessels. Finally, acinar cells were characterized by their basal basophilia and apical acidophilia.

Histological examination revealed that islet blood vessels were dilated in alcohol-induced pancreatitis mice (Figure 1B,C). The pancreas of the LA and MA groups, in contrast, displayed abnormal tissue architecture with distinct areas of acinar cell loss and pseudotubular structures. Importantly, the areas of acinar cell injury in the pancreas of alcohol-fed mice were significantly greater than in the C group. When acinar cell loss was assessed, the average area of acinar cell injury was nearly double in the pancreas of the LA and MA groups compared to the C group (Figure 1B,C).

β-carotene was associated with an increase in the number and size of pancreatic islets, as shown in Figure 1D–F. The islets had a lobular aspect, a regular shape, and some fibrotic growth in them. In comparison with groups without β-carotene supplementation, the exocrine pancreas had less cell degeneration and vacuolization, but had eosinophilic cytoplasm.

Table 2 shows the percentages of animals that were categorized to each score of the two histological parameters. The median (lower and upper range) of Ammann’s fibrosis score for C was 2.36 (2–4), for LA was 6.44 (6–8), for MA was 10.52 (10–12), for B was 3.84 (2–5), for LA + B was 8.04 (6–10), and for MA + B was 3.88 (2–5). Analysis of peri-lobular parenchyma, intralobular parenchyma, and total score for Ammann’s fibrosis punctuation in the pancreas found significant differences between groups (*p* < 0.001; Table 3).

### 2.3. Morphoquantitative Analysis of the Pancreas

The histological changes in pancreatic islet mass can be coupled with clear qualitative differences in overall cytology, with islets appearing small and well defined in the C, LA, and LA + B groups (Figure 1A,B,E).

In the pancreas of mice exposed to alcohol intake and/or β-carotene supplementation, the N_V_ and V_V_ values for pancreatic islets were higher in the LA + B and MA + B groups in comparison with the LA and MA groups, respectively (Table 4). The N_V_ of acinar cells decreased in the MA + B group in comparison with the MA group (*p* = 0.245). Post hoc tests showed statistically significant differences in the N_V_ and V_V_ of pancreatic islets (*p* < 0.001) and the V_V_ and S_V_ of acinar cells (*p* < 0.014).

## 3. Discussion

### 3.1. Summary of Key Findings and Interpretation

Chronic pancreatitis is distinguished by the presence of fibroinflammatory alterations in the pancreatic tissue. The development of this disorder can be observed in conjunction with several factors, including alcohol misuse, smoking, gene mutations, autoimmune syndromes, metabolic changes, environmental conditions, and anatomical anomalies [33,34,35].

Although acute pancreatitis is a well-recognized cause of hospitalization, accurately diagnosing this condition can be challenging due to the absence of a reliable and straightforward blood test that exhibits high sensitivity. The indicators that are often utilized include serum amylase, lipase, trypsinogen-2, and activation peptide of carboxypeptidase B [36,37,38].

### 3.2. Biochemical Evaluation

Standard biochemical indicators employed in clinical settings encompass serum amylase and lipase. However, amylase levels in the serum normalize within 3–5 days, while lipase levels typically normalize between 8 and 14 days [39]. The presence of increased levels of amylase and/or lipase in the blood indicates a higher likelihood of acute pancreatitis, with amylase being the more commonly utilized measurement [38]. Approximately 40% of serum amylase originates from the pancreas, while the majority comes from the salivary glands [40]. Hence, an increase in serum total amylase levels is not exclusive to pancreatitis, and it is important to evaluate other illnesses as well [40]. The L/A ratio is an acceptable indicator of alcohol-induced pancreatitis [41]. In fact, L/A ratio can be used to distinguish between pancreatitis caused by alcohol use and pancreatitis not caused by alcohol [42]. Patients diagnosed with alcohol-induced pancreatitis have a twofold increased likelihood of having an L/A ratio of three or higher with a sensitivity of 75% and a specificity of 56% [43]. Our results show increased lipase levels in the B and LA + B groups in comparison to the C group (*p* = 0.002). In addition, decreased lipase levels and L/A ratio in the MA + B group were found (*p* = 0.002 and *p* < 0.001, respectively). No significant differences in amylase levels were found (*p* = 0.155). These results suggest that β-carotene supplementation prevents acute pancreatitis caused by alcohol and improves pancreas function after moderate alcohol consumption, and they could be an acceptable indicator of alcohol-induced pancreatitis.

Acinar cells in cell culture or isolated pancreatic acini cells have been shown to break down ethanol in two ways: one is by oxidation, and the other is by non-oxidation [44,45,46]. When it comes to the oxidative pathways, the most common form (called an isoform) of ADH in acinar cells is ADH3. This is an enzyme that cannot reach saturation and has a low affinity for ethanol and a high Km value. CYP2E1 has been identified in both the human and rat pancreas. As noted before, the expression of this gene can be stimulated in the pancreas of rats that have been fed alcohol [47].

Prolonged exposure to ethanol in mice leads to an increase in aldehyde dehydrogenase (ALDH) activity, suggesting the development of physiological tolerance [48]. However, animals who were exposed to long-term consumption of ethanol and received oral administration of β-carotene showed increased levels of ALDH in their bloodstream. In contrast, the groups that did not receive supplementation exhibited a decrease in the activity of this enzyme [49]. Previous research has confirmed that individuals suffering from chronic liver disease demonstrate diminished ALDH activity in comparison to individuals who do not have the ailment [48]. Multiple studies have noted a substantial decrease in overall ALDH levels among individuals who have progressed to severe liver disease. The decrease is especially evident in individuals with elevated levels of fibrosis and liver damage [50].

### 3.3. Histological Evaluation

The cardinal histopathologic features of chronic pancreatitis are fibrosis, loss of acinar tissue, and ductal changes [43]. The fibrosis score has been widely utilized in numerous studies to evaluate fibrosis [51]. The evaluation approach initially examines whether perilobular fibrosis is localized or widespread and subsequently categorizes the perilobular fibrosis into one of three degrees: mild, moderate, or severe.

The findings strongly indicate that supplementing mice with β-carotene can ameliorate the pancreatitis associated with prolonged ethanol exposure. The proposed treatment appeared to be more efficacious in animals with moderate alcohol consumption than in the low-alcohol-consumption group. Chronic alcohol intake is responsible for 17% to 25% of acute pancreatitis cases globally and ranks as the second most prevalent cause of acute pancreatitis, following gallstones [52]. Research conducted in recent years has provided evidence indicating that the causes of steatosis induced by ethanol are likely to be complex, involving various factors such as the impact on liver lipid metabolism, hypoxia, oxidative stress, pancreas function, and lipid peroxidation [21,49,53,54]. In accordance with this, our results showed dilated blood vessels in islets, abnormal tissue architecture with distinct areas of acinar cell loss, and pseudotubular structures in mice fed low and moderate alcohol doses (Figure 1B,C). These results agree with previous studies, where long-term ethanol treatment induces pancreatic islet dysfunction and apoptosis [55].

Fruits and vegetables contain large amounts of antioxidant micronutrients, such as vitamins and carotenoids. These micronutrients play a role in protecting the organism against reactive oxygen species [20]. Studies have demonstrated a reduction in antioxidant vitamins and carotenoids in several pancreatic illnesses, leading to a decreased likelihood of pancreatic neoplasia [56,57] and pancreatic cancer [58]. We demonstrated that β-carotene supplementation improved the number, size, and shape of pancreatic islets in alcohol-induced pancreatitis mice with some fibrotic growth (Figure 1D–F). In addition, analysis of peri-lobular parenchyma, intra-lobular parenchyma, and the total score for Ammann’s fibrosis punctuation in the pancreas found that the LA + B and MA + B groups showed differences to the C group, while the MA + B group showed differences to the B group (*p* < 0.001; Table 3). At the final assessment, it was discovered that the levels of beta-carotene were significantly elevated in patients with moderate acute pancreatitis compared to those with severe acute pancreatitis [59]. The association between decreased antioxidant levels and increased disease severity indicates the effectiveness of antioxidant supplementation therapy [60].

### 3.4. Morphoquantitative Analysis of the Pancreas

Pancreatitis has multiple causes, with alcohol and gallstones being the most prevalent etiologies. The exact underlying mechanisms of this disease are not fully understood; nonetheless, it is probable that it originates from the effects of alcohol on the small pancreatic ducts and acinar cells. Alcohol is believed to cause the formation of protein plugs in the narrow channels of the pancreas by increasing the thickness of pancreatic secretions. The plugs subsequently harden into calculi, resulting in progressive inflammation and fibrosis [4]. Acinar, islet, and ductal cells are lost in the end because of this process [4,61]. Based on our findings, it can be deduced that the ingestion of β-carotene orally counteracts the consequences of prolonged alcohol intake in the pancreatic ducts and acinar cells (Table 4). This accounts for the variations observed in the stereological examination of N_V islets_, V_V islets_, and TM _islets_ (*p* < 0.001, *p* < 0.003 and *p* < 0.001; respectively), but not in N_V acinar cells_, V_V acinar cells_, or S_V acinar cells_ (*p* = 0.994, *p* = 0.868 and *p* = 0.987, respectively) between the MA + B and C groups. In fact, several in vivo and in vitro investigations have demonstrated that antioxidants can effectively suppress pancreatic fibrosis [62,63,64], where glutathione is a prominent antioxidant found within cells, which serves a crucial function in mitigating the impact of oxidative stress [65].

### 3.5. Scope and Limitations

The purpose of this study was to ascertain what happened to the biochemical parameters and pancreatic histology of *C57BL/6* mice that received oral beta-carotene after ethanol consumption. Our data provide new evidence of the connection between alcohol-induced pancreatitis and antioxidant therapies, such as β-carotene. Still, this study has some limitations: 1. It does not look at any connections between alcohol metabolism byproducts that are not oxidative, specifically fatty acid ethyl esters, or antioxidant treatments; 2. It does not look at any autophagy- or inflammation-related endpoints; 3. It needs more experiments to look at the substances that hurt pancreatic acinar cells; and 4. It needs collagen immunocytochemistry to confirm the short-term damage caused by heavy alcohol use. These aspects should be addressed in future investigations. However, our data suggest that exposure to β-carotene decreased pancreatic damage during moderate alcohol exposure in *C57BL/6* mice.

## 4. Materials and Methods

### 4.1. Animals

Thirty-six male *C57BL/6* mice (*Mus musculus*) aged fifty days were obtained from the Public Health Institute of Chile. In order to help them adjust to their new environment, they were housed for 30 days in the animal facility of the Center of Excellence in Morphological and Surgical Studies (CEMyQ) at the Universidad de La Frontera. Standard laboratory food (AIN-93M) [66] and water were provided, and they were kept in a 12 h light/dark cycle (08:00–20:00/20:00–08:00). All of the mice were given a normal laboratory diet (AIN-93M) during the experimentation period in accordance with the guidelines set forth by the Institute for Laboratory Animal Research’s Committee for the Update of the Guide for the Care and Use of Laboratory Animals [67].

On the first day of the experiment (Day 1), the mice were divided into six groups: 1. Group C (control); 2. Group LA (low-dose alcohol): 3% *v*/*v* ad libitum alcohol administration for 28 days [68]; 3. Group MA (moderate-dose alcohol): 7% *v*/*v* ad libitum alcohol administration for 28 days [68]; 4. Group B (β-carotene): 0.52 mg/kg body weight/day β-carotene administration for 28 days [69]; 5. Group LA + B (Low-dose alcohol + β-carotene): low-dose alcohol plus administration of 0.52 mg/kg body weight/day of β-carotene for 28 days; and 6. Group MA + B (moderate-dose alcohol + β-carotene): moderate-dose alcohol plus administration of 0.52 mg/kg body weight/day β-carotene for 28 days. Body mass was measured at the beginning, during the whole experimental phase, and at the end of the experimental phase. The results are shown in our previous study [49].

The modified liquid diet of Lieber–DeCarli was followed for the administration of oral chronic ethanol in drinking water [68,70]. An oral dose of 0.52 mg/kg body weight/day of β-carotene was given [69]. The experimental model has been used and tested previously [23,54].

### 4.2. Euthanasia

At the end of the experiment on Day 28, the animals were fasted for 6 h and euthanized with sodium pentobarbital.

### 4.3. Biochemistry

For the serum analyses, the serum was isolated using centrifugation at a speed of 2058× *g* for a duration of 15 min. The isolated serum was then stored at a temperature of −80 °C until it was ready for analysis. The lipase and amylase enzymes (Life Technologies, Thermo Fisher Scientific Inc., Waltham, MA, USA) were used to look at the pancreatic physiology. Furthermore, the quantification of uric acid concentration was performed using a colorimetric kit from Life Technologies, Thermo Fisher Scientific Inc., located in Waltham, MA, USA.

### 4.4. Processing and Staining of Pancreas

Considering the isotropic properties of the tissue, many sections of each pancreas were obtained to obtain representative characteristics for them. When they were dehydrated, they were embedded in Paraplast Plus (Sigma-Aldrich Co., Ltd., St. Louis, MO, USA). This was done after they had been fixed for 48 h at 4% in buffered formalin (1.27 mol·L^−1^ of formaldehyde in 0.1 M phosphate buffer, pH 7.2). After obtaining the blocks, a microtome (Leica^®^ RM2255, Leica^®^, Wetzlar, Germany) was used to make cuts that were 5 μm thick. Each block was then divided into five pieces, each of which was stained with hematoxylin and eosin (H&E) for histological analysis.

### 4.5. Histological Evaluation

Histologic grading was conducted by researchers who had received specialized training using Ammann’s fibrosis score [51]. The researcher examined the slides in isolation, without any awareness of the research groups. Fibrosis in the peri-lobular and intralobular parts of the parenchyma was scored using Ammann’s Fibrosis System, which runs from 1 to 6. These two elements add up to the final score. The evaluated parameters are shown in Table 5.

### 4.6. Morphoquantitative Analysis of the Pancreas

Five animals from each group were used for the stereological investigation. Using an analytical balance and Scherle’s approach, the mass and volume of the pancreas were ascertained [71]. We took five different pieces from each pancreas, dried them, and then embedded them in Paraplast Plus (Sigma-Aldrich Co., Ltd., St. Louis, MO, USA). This was done after 48 h of treatment with 4% buffered formalin (1.27 mol/L of formaldehyde in phosphate buffer 0.1 M, pH 7.2). After obtaining the blocks, 5 μm thick slices were created and stained with H&E.

Ten fields were observed for each region in the stereological research, for a total of 50 fields per group [72,73]. A stereological microscope (Leica^®^ DM2000 LED, Leica^®^, Wetzlar, Germany) was used to view the slides, and a digital camera (Leica^®^ MC170 HD, Leica^®^, Wetzlar, Germany) was used to take pictures. Using the STEPanizer^®^ 36-point test method (STEPanizer^®^, Version 1, Software Eng., Institute of Anatomy, University of Bern, Switzerland), it was possible to determine the pancreatic islets’ number density (N_V islets_), volume density (V_V islets_), and surface density (S_V islets_). Furthermore, the pancreatic islets’ total mass (TM _islets_) was ascertained. The N_V islets_ were measured using the following formula: N_V islets_ are equal to Q-/(A_T_ × t), where A_T_ is the entire area of the test system and dissector thickness (*t*) and Q- is the number of observations in a certain area considering the banned lines and the prohibited plane. The formula used to estimate V_V islets_ was V_V islets_ = P_P islets_/P_T_ (100%), where P_T_ is the total number of points in the system and P_P islets_ is the number of points that touch the pancreatic islets. The S_V islets_ were calculated using the following equation: S_V islets_ are equal to (2 × I)/L_T_, where L_T_ is the total length of the lines in the 36-point test system, and I is the number of intersections that touch the structure. The estimation of N_V_, V_V_, and S_V_ acinar cells was done in accordance with earlier instructions. By multiplying V_V islets_ by the mass of the pancreas, TM _islets_ was calculated.

### 4.7. Statistical Analysis

Levene’s test (homoscedasticity of the variances) and the Kolmogorov–Smirnov test (analysis of data normalcy) were used to assess differences in the quantitative data. A one-way ANOVA was used to assess the group differences, and, if necessary, the Dunnett’s T3 test or Tukey’s post-hoc HSD test was then performed. According to IBM Corp., Armonk, NY, USA, *p* < 0.05 was deemed statistically significant (IBM SPSS Statistics, Version 21, Endicott, NY, USA).

## 5. Conclusions

In our study, biochemical parameters changed significantly in the MA + B group compared to the C, MA, B, and LA + B groups. Specifically, serum total amylase levels went up and lipase levels went down. In addition, the histological study showed that peri-lobular parenchyma, intralobular parenchyma, and fibrosis score were less punctuated in the MA + B group than in the LA, MA, and LA + B groups. Examination of the islets and acinar cells of the pancreas stereologically after the drinking of moderate amounts of alcohol and β-carotene also showed positive results. These findings suggest that antioxidant therapies may be beneficial in treating ethanol exposure in animal models. Although our understanding of the mechanisms behind antioxidant supplements is extensive, further investigation is required to explore the relationship between alcohol consumption and antioxidant therapies. This entails conducting research using specific cell lines and clinical trials to delve into the signaling pathways as well as the enzyme and non-enzyme mechanisms involved.

## Figures and Tables

**Table 1:** Biochemical analysis of male *C57BL/6* mice exposed to alcohol consumption and oral β-carotene supplementation.

Media ± SD							
	C	LA	MA	B	LA + B	MA + B	*p*
Lipase (U/L)	51.315 ± 7.230	43.363 ± 3.377 ^a^	55.975 ± 14.098	65.383 ± 6.679 ^ab^	64.315 ± 9.555 ^ab^	47.139 ± 6.099 ^de^	0.002
Uric acid (μmol/L)	99.511 ± 29.729	124.666 ± 24.118	59.983 ± 18.445 ^ab^	104.487 ± 28.909 ^c^	91.219 ± 26.863 ^bc^	113.609 ± 14.520 ^ce^	<0.001
Amylase (U/L)	658.420 ± 195.988	705.070 ± 95.701	712.886 ± 134.047	709.438 ± 125.675	735.407 ± 123.837	825.953 ± 193.412	0.155
Lipase/Amylase ratio	0.081 ± 0.018	0.062 ± 0.011	0.085 ± 0.041	0.096 ± 0.027 ^b^	0.096 ± 0.021 ^b^	0.058 ± 0.011 ^de^	<0.001

^a^ Significant differences (*p* < 0.05) with the C group. ^b^ Significant differences (*p* < 0.05) with the LA group. ^c^ Significant differences (*p* < 0.05) with the MA group. ^d^ Significant differences (*p* < 0.05) with the B group. ^e^ Significant differences (*p* < 0.05) with the LA + B group.

**Table 2:** Percentages of the histological changes according to Ammann’s fibrosis punctuation.

Parameters	Score	Fibrosis Grade	Group Frequency (%)					
			C	LA	MA	B	LA + B	MA + B
Peri-lobular parenchyma	1	Mild	80	0	0	16	0	20
	2	Moderate	20	0	0	76	0	76
	3	Marked	0	80	0	8	20	4
	4	Mild	0	20	0	0	64	0
	5	Moderate	0	0	72	0	16	0
	6	Marked	0	0	28	0	0	0
Intralobular parenchyma	1	Mild	84	0	0	20	0	16
	2	Moderate	16	0	0	68	0	64
	3	Marked	0	76	0	12	20	20
	4	Mild	0	24	0	0	52	0
	5	Moderate	0	0	76	0	28	0
	6	Marked	0	0	24	0	0	0

**Table 3:** Analysis of peri-lobular parenchyma, intralobular parenchyma, and total score for Ammann’s fibrosis punctuation in pancreas.

Media ± SD							
	C	LA	MA	B	LA + B	MA + B	*p*
Peri-lobular parenchyma	1.200 ± 0.408	3.200 ± 0.408 ^a^	5.280 ± 0.458 ^ab^	1.920 ± 0.493 ^abc^	3.960 ± 0.611 ^abcd^	1.840 ± 0.472 ^abce^	<0.001
Intralobular parenchyma	1.160 ± 0.374	3.240 ± 0.435 ^a^	5.240 ± 0.435 ^ab^	1.920 ± 0.571 ^abc^	4.080 ± 0.702 ^abcd^	2.040 ± 0.611 ^abce^	<0.001
Score	2.360 ± 0.568	6.440 ± 0.711 ^a^	10.520 ± 0.714 ^ab^	3.840 ± 0.850 ^abc^	8.040 ± 1.01 ^abcd^	3.880 ± 0.781 ^abce^	<0.001

^a^ Significant differences (*p* < 0.05) with the C group. ^b^ Significant differences (*p* < 0.05) with the LA group. ^c^ Significant differences (*p* < 0.05) with the MA group. ^d^ Significant differences (*p* < 0.05) with the B group. ^e^ Significant differences (*p* < 0.05) with the LA + B group.

**Table 4:** Stereological analysis of mice pancreas exposed to alcohol consumption and oral supplementation of β-carotene.

Media ± SD							
	C	LA	MA	B	LA + B	MA + B	*p*
N_V islets_ (mm^−3^)	429.183 ± 52.588	604.177 ± 20.168 ^a^	651.080 ± 51.059 ^a^	672.110 ± 85.790 ^a^	715.472 ± 69.053 ^a^	707.522 ± 31.115 ^a^	<0.001
V_V islets_ (%)	23.053 ± 7.539	28.307 ± 7.251	36.167 ± 8.927 ^a^	27.595 ± 2.455	28.547 ± 4.488	38.386 ± 8.445 ^a^	0.001
S_V islets_ (mm^−1^)	7.011 ± 0.219	9.626 ± 1.093	10.077 ± 1.122	9.370 ± 3.840	9.370 ± 3.511	9.182 ± 6.373	0.144
TM _islets_	3.270 ± 0.028	4.092 ± 0.052	6.862 ± 0.639 ^ab^	6.208 ± 0.701 ^a^	4.984 ± 0.460	8.398 ± 0.633 ^abd^	<0.001
N_V acinar cells_ (mm^−3^)	2476.634 ± 854.875	2529.968 ± 890.316	3030.235 ± 283.025	2668.711 ± 907.537	2710.377 ± 466.376	2296.665 ± 756.358	0.245
V_V acinar cells_ (%)	4.872 ± 1.052	5.228 ± 1.988	4.537 ± 0.295	3.904 ± 0.267 ^b^	5.014 ± 0.340	5.452 ± 0.380 ^c^	0.014
S_V acinar cells_ (mm^−1^)	15.266 ± 6.842	15.994 ± 2.595	13.589 ± 3.246	12.043 ± 3.546 ^b^	13.698 ± 1.666	14.439 ± 1.017	0.014

N_V islets_: number density per area of the pancreatic islets; V_V islets_: volume density per area of the pancreatic islets; S_V islets_: surface density per area of the pancreatic islets; TM _islets_: total mass of the pancreatic islets; N_V acinar cells_: number density per area of the acinar cells; V_V acinar cells_: volume density per area of the acinar cells; S_V acinar cells_: surface density per area of the acinar cells. ^a^ Significant differences (*p* < 0.05) with the C group. ^b^ Significant differences (*p* < 0.05) with the LA group. ^c^ Significant differences (*p* < 0.05) with the B group. ^d^ Significant differences (*p* < 0.05) with the LA + B group.

**Table 5:** The scoring system for histological changes according to Ammann’s fibrosis punctuation.

Score	Fibrosis Grade	Peri-Lobular Parenchyma	Intralobular Parenchyma
1	Mild	Lobules are separated by fibrous tissue without any changes in structure or atrophy.	Thin fibrous threads that separate the acini within the lobules, but without any substantial changes to the overall structure.
2	Moderate	Lobules are separated by fibrous tissue with changes in structure or atrophy (between 0 and 20%).	Fibrous threads that separate the acini within the lobules, with substantial changes to the overall structure (between 0 and 20%).
3	Marked	Lobules are separated by fibrous tissue with changes in structure or atrophy (between 20 and 40%).	Fibrous threads that separate the acini within the lobules, with substantial changes to the overall structure (between 20 and 40%).
4	Mild	Lobules are separated by fibrous tissue with changes in structure or atrophy (between 40 and 60%).	Fibrous threads that separate the acini within the lobules, with substantial changes to the overall structure (between 40 and 60%).
5	Moderate	Lobules are separated by fibrous tissue with changes in structure or atrophy (between 60 and 80%).	Fibrous threads that separate the acini within the lobules, with substantial changes to the overall structure (between 60 and 80%).
6	Marked	Lobules are separated by fibrous tissue with changes in structure or atrophy (between 80 and 100%).	Fibrous threads that separate the acini within the lobules, with substantial changes to the overall structure (between 80 and 100%).

## Data Availability

The original contributions presented in the study are publicly available. This data can be found here: https://doi.org/10.6084/m9.figshare.24988047.v1.
